# Degradation Parameters from Pulse-Chase Experiments

**DOI:** 10.1371/journal.pone.0155028

**Published:** 2016-05-16

**Authors:** Celine Sin, Davide Chiarugi, Angelo Valleriani

**Affiliations:** Department of Theory and Bio-Systems, Max Planck Institute of Colloids and Interfaces, D-14424 Potsdam, Germany; University Paris South, FRANCE

## Abstract

Pulse-chase experiments are often used to study the degradation of macromolecules such as proteins or mRNA. Considerations for the choice of pulse length include the toxicity of the pulse to the cell and maximization of labeling. In the general case of non-exponential decay, varying the length of the pulse results in decay patterns that look different. Analysis of these patterns without consideration to pulse length would yield incorrect degradation parameters. Here we propose a method that constructively includes pulse length in the analysis of decay patterns and extracts the parameters of the underlying degradation process. We also show how to extract decay parameters reliably from measurements taken during the pulse phase.

## Introduction

**Fig 1:**
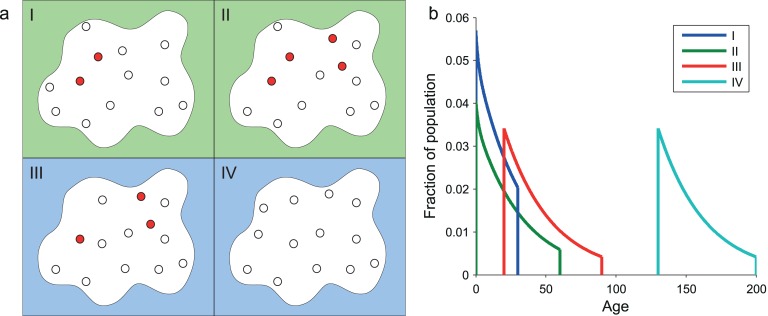
Age distribution of molecules during pulse chase experiments. **(a)** Depiction of molecules in a cell during a pulse chase experiment with pulse duration of 70 in arbitrary units (a.u.). For the purpose of illustration we show four snapshots of the experiment. In snapshot I (30 a.u.), pulse has just begun. The white dots depict the population of molecules already existing in the cell before the pulse. All newly synthesized molecules (red dots) are labeled by the pulse and measurable by the experimentalist. As the pulse continues in snapshot II (60 a.u.) we see more labeled molecules appear. Meanwhile, both labeled and unlabeled molecules degrade. In snapshot III (90 a.u.), the pulse has ended since some time. Newly synthesized molecules from this moment on are unlabeled. Again, both labeled and unlabeled molecules degrade. In snapshot IV (200 a.u.), all labeled molecules have degraded. Unlabeled molecules continue to be synthesized and degraded. **(b)** Age distribution of the labeled molecules, each curve corresponds to one phase in panel (a). In snapshot I, the pulse has just begun, and all molecules that are labeled by the pulse are no older than the time elapsed since the pulse has begun. In snapshot II, the pulse has been applied for some time; some labeled molecules may be quite old. In snapshot III the molecules cannot be younger than the time elapsed since pulse has been stopped. By snapshot IV, if there were molecules left, they would have that age distribution.

The degradation of macromolecules such as mRNA and proteins is a complex biochemical process that is usually carried out by a series of subsequent biochemical reactions. Various experimental techniques can be used to study the decay of macromolecules over time; one of the most widespread methods to measure decay is pulse-chase experiments. A pulse-chase experiment consists of two phases: first, in the pulse phase, cells are exposed to a labeling compound that is integrated into newly synthesized macromolecules of interest. For example, if one wants to follow the fate of proteins or mRNA radio or isotope labeled amino acids [1–5] or nucleotides [6, 7] can be introduced in the culture medium. In the second phase, the chase phase, the same compound in the unlabeled form is added in excess, replacing the labeled form. Any macromolecules synthesized during the chase phase will not be labeled. Throughout the chase, the amount of macromolecules that were synthesized during the pulse diminish due to degradation processes (Fig 1a). Tracking their amount over time delivers a curve that we call the *decay pattern*. Alternative methods, such as stopping synthesis of the macromolecule and measuring the amount left over time, also provide decay patterns. For example, chloramphenicol is often used to stop protein synthesis in bacterial cells. However, such methods may be very stressful for the cell, potentially resulting in measurements not indicative of normal cell conditions. Ultimately, the process of stopping synthesis corresponds in general to a pulse of infinite duration. From the point of view of data analysis, decay patterns obtained upon stopping the synthesis are a limit case of patterns generated with pulse-chase experiments.

Decay patterns are said to be exponential, if they look like straight lines in a plot linear in time and logarithmic in the (relative) abundance of the molecule. This makes fitting a single exponential to the decay pattern especially convenient—so much so that it is often used as the default procedure for analysis of decay curves. Unfortunately this procedure is often overused; one can tell when a quick look at the plot clearly indicates that the pattern is not as straight as it ought to be. As a way out of this cumbersome situation, many research studies use the half-time as a measure of the stability of the molecule because it can be estimated even without any fit of the data. For exponentially decaying molecules, there is a simple algebraic relationship between half-time and average lifetime of the molecules, but this relationship does not hold for non-exponentially decaying molecules. Thus, half-time is not a good measure of stability for non-exponential decay [8]. A further complication is that apparently, for some decay patterns, the pulse length affects the half-time [9]. This happens because the half-time is measured by simply extracting it from the data without taking pulse length into account. Thus, the half-time of the resultant population is actually not a quantity representative of the molecule’s decay alone, but also of the pulse length. The choice of the function used for the analysis of decay curves makes critical assumptions about the biochemistry of the degradation process at the single molecule level. As we shall see, the choice of the exponential function may lead to achieve incorrect information about the underlying biochemical processes and the stability of the macromolecules.

Another common approach to analyze non-exponential (sometimes called nonlinear) decay patterns is to fit them with a double exponential. Although this certainly improves the fit, it is not yet immediately obvious how the two characteristic timescales from the two exponentials depend on the length of the pulse or on other possible choices of the experimental design.

One fundamental question is: What is the process behind the decay pattern? We know that behind the decay pattern there is a degradation process. When we think about the degradation process, we think at the level of single molecules. Namely, we think at the processes (biochemical networks, perhaps competing against each other) that will eventually lead to the disappearance of the molecule. Taking into account that we are talking about biochemical interactions, the fate of each single molecule can be described quantitatively only in probabilistic terms, with the basic quantity of this description being the probability distribution of the molecule’s lifetime. The decay pattern, however, is not a single molecule measurement. Rather, it is the result of billions of molecules, each randomly decaying during and after the pulse.

When pulse-chase experiments are used to follow the macromolecules degradation, a bias may be introduced into the measurements through the pulse length. In this paper we will show that the length of the pulse affects the shape of the decay patterns. If this effect is not taken into consideration, the biochemical information we extract from the decay pattern would be biased. Unfortunately, this effect is usually not considered when choosing the pulse length. The connection between the single molecule perspective and the decay pattern requires an abstract view of the degradation process. Here we present an approach that allows us to correctly bridge the gap between the single molecule perspective and the population level for a wide range of decay patterns. Notably, our method provides a simple recipe that allows us to incorporate the pulse length in the fitting procedure when decay patterns are derived from pulse-chase experiments. The method does not rely on any *a priori* knowledge of the lifetime of the molecule and can be used independently of whether the pulse length is short or long compared to the lifetime of the molecules. First, we recapitulate the general theory providing a new derivation of the required mathematics and then we provide a simple recipe to incorporate the pulse length in the fitting procedure. Our results should be applied to refine the protocols for the design of decay assay experiments.

### Aging of molecules

It is rather strange that an apparently simple concept like aging becomes so intriguing when applied to molecules. Molecules, at least complex molecules like proteins and mRNA, age in a fashion which is very similar to what we know based on our daily experience. Damage, shortening or lengthening, the attachment of other molecules (e.g. miRISC complex that attaches to the mRNA [10, 11]) are common phenomena that make the target molecule older. In fact, some processes could even make the target molecule younger, e.g. when damage is repaired or a bound molecule detaches. What does it actually mean when a molecule becomes older, or ages? How can we characterize this phenomenon? Acutally, what best characterizes the effect of aging is not how much time has passed since birth or synthesis but the amount of time left until the end of life.

Let us look at this matter closely. Suppose for a moment that the lifetime distribution of an hypothetical molecule is exponential—as we shall see later, this implies that also the decay pattern is exponential—and we know that in this precise moment the age of the molecule is *a*, *i.e.* this molecule has been synthesized *a* time units ago. What is the distribution of its residual lifetime? As a consequence of the assumption of an exponential lifetime distribution, the answer is that the residual lifetime is independent of *a* and is the same as if the molecule had been synthesized right now. This answer means that the molecule does not age. However, if we know or suspect that molecules like mRNA and proteins must undergo a series of biochemical reactions [12–14] until we consider them as degraded, each step in the biochemical reaction network makes the molecule older, *i.e.* closer to its final end. When the molecule ages in the “complex” way just described, its residual lifetime becomes shorter. Therefore, its lifetime distribution cannot be exponential and so also the decay pattern cannot be an exponential.

Once we accept that molecules age—*i.e.* do not have an exponential lifetime distribution—there is another effect of aging related to the duration of the pulse. Imagine a pulse of a very short duration. The molecules synthesized during the pulse will all have quite the same residual lifetime, since they are likely to be all in exactly the same biochemical state. When the pulse becomes longer, some of the molecules synthesized at the beginning of the pulse may have been already degraded, some are definitively older but still there, and others will be just newly synthesized. In short, we have a mixture of ages in the population, and the composition of the population depends on the pulse length (Fig 1b). This is an important point for what follows: the initial condition at the time point of chase depends on the mixture of ages in the population of molecules, which depends on the length of the pulse. Therefore, fitting the decay pattern requires a knowledge of the age distribution at the beginning of the chase, which can be computed only if one knows the effect of the pulse on the age distribution. The effect of the pulse on the age distribution can only be known if one has a good model to describe how the molecules age, and has already calibrated the model. A priori, it is not possible to know if a pulse of a given length is long or short compared to the lifetime of the molecules, but as we shall see, this apparent circularity can be solved to provide a unique formula that takes pulse, chase and aging into account.

### Degradation processes modeled with Markov chains

Macromolecules are degraded through a number of different pathways. While some can be approximated as exponentially decaying, many of these pathways are multi-step pathways with several complex biochemical stages [1, 12–15]. The biochemical stages are connected to each other to form a network of biochemical states [8, 16–18]. When we describe the dynamics of degradation of a single molecule, we think of this molecule as moving on this network of biochemical states in a stochastic fashion until degradation eventually happens. We will use the term “single step degradation” for pathways with only one measurable rate limiting step (*i.e.* exponentially decaying), and the term “multi-step degradation” for more complex pathways, which necessarily includes the “single step” degradation as a limiting case [8]. The rates of the transitions between the biochemical states would depend on the concentration of the ligands and on their affinity to the target molecule, details rarely available on a large scale. It is therefore convenient to model this process as a Markov chain, which is a simple and mathematically treatable tool to describe stochastic process on discrete states [8, 16, 17, 19–21]. From this we can model the decay as a single molecule stochastic process, and then solve the equations to derive the lifetime distribution, the age-dependent degradation rate, and the steady state distribution of the fraction of molecules in each state.

## Methods

If *T* is the random variable describing the lifetime of a molecule, we define *f*_*T*_(*t*) its probability density function. This definition means that

$$\begin{array}{c} \mathrm{Pr}\left\{T\leq t\right\}\;=\;\int_{0}^{t}f_{{}_{T}}\left(u\right)du\;\equiv \;F_{{}_{T}}\left(t\right)\;, \end{array}$$ (1)

with *F*_*T*_ being the probability distribution. For later use, we state here also the equation for the average lifetime

$$\begin{array}{c} \bar{T}\;=\;\int_{0}^{\infty }\;uf_{{}_{T}}\left(u\right)du\;=\;\int_{0}^{\infty }\left(1-F_{{}_{T}}\left(u\right)\right)du\;. \end{array}$$ (2)

The age-dependent degradation rate *δ*(*a*) is defined as the probability that a molecule of age *a* is degraded in the next unit of time. Formally, it is related to the lifetime probability density *f*_*T*_ by means of a relationship known in the literature as the definition of hazard rate:

$$\begin{array}{c} \delta \left(a\right)\;=\;\frac{f_{{}_{T}}\left(a\right)}{1-F_{{}_{T}}\left(a\right)}\;, \end{array}$$ (3)

which is a tool commonly employed in survival analysis [22]. When *T* is exponentially distributed with rate *μ*, then *δ*(*a*) is a constant equal to *μ*. When, however, *f*_*T*_ is not exponential then *δ*(*a*) can take many different forms, the most common of which are the monotonously decreasing to a constant larger than zero (molecules stabilize with age) and increasing (molecules destabilize with age). The results presented in this manuscript concern with the derivation of *f*_*T*_ from a model and the fit of this model with the available data, *i.e.* the decay patterns. In some cases, however, it is possible to first formulate a specific form of the age-dependent degradation rate *δ*(*a*), based on some specific model of degradation, and then derive the lifetime density as

$$\begin{array}{c} f_{{}_{T}}\left(t\right)\;=\;\delta \left(a\right)\exp\left(-\int_{0}^{t}\delta \left(a\right)da\right)\;, \end{array}$$ (4)

and its probability distribution as

$$\begin{array}{c} F_{{}_{T}}\left(t\right)\;=\;1\;-\;\exp\left(-\int_{0}^{t}\delta \left(a\right)da\right)\;. \end{array}$$ (5)

An example of a derivation that starts with the hazard rate was given in Ref. [20].

### Decay after a pulse

Consider a system that starts with zero molecules, so that *N*(0) = 0. Through the process of synthesis with constant rate and through a generic complex degradation process, the average number of molecules *N*(*t*) will first increase in time and eventually reach a steady state. To derive the full pattern *N*(*t*) consider dividing the time *t* into *k* small intervals each of duration *τ* so that *kτ* = *t*. Given a constant synthesis rate *ω*, we have that *N*(*τ*) = *ωτ*(1 − *F*_*T*_(*τ*)). At the next time interval, we have that *N*(2*τ*) = *ωτ*(1 − *F*_*T*_(2*τ*)) + *ωτ*(1 − *F*_*T*_(*τ*)), where the first term gives the probability that the molecules synthesized in the first interval survive until the end of the second interval. Proceeding in this way one can verify that *N*(*kτ*) = *N*(*kτ* − *τ*) + *ωτ*(1 − *F*_*T*_(*kτ*)). Letting now *k* → ∞ and *τ* → 0 while keeping *kτ* = *t*, leads to the differential equation

$$\begin{array}{c} \frac{dN(t)}{dt}\;=\;\omega \left(1-F_{T}\left(t\right)\right)\;, \end{array}$$ (6)

from which it follows that

$$\begin{array}{c} N\left(t\right)\;=\;\omega \int_{0}^{t}\left(1-F_{{}_{T}}\left(u\right)\right)du\;. \end{array}$$ (7)

*N*(*t*) is thus the amount of molecules present at time *t* when their origination time was between 0 and *t*. Therefore, if pulse or synthesis is not stopped, when *t* goes to infinity the average number of molecules reaches a steady state value where synthesis and degradation balance each other.

At any time, the number of new molecules delivered after an interval Δ*t* of synthesis is thus given by *N*(Δ*t*). Consider now the quantity *N*(*t* + Δ*t*) − *N*(*t*). If synthesis works normally, this quantity must be non negative and has two contributions: the molecules delivered at time *t* that survived until *t* + Δ*t* plus the newly synthesized molecules that have been delivered after an interval Δ*t*, which is given by *N*(Δ*t*). Therefore, if *N*(*t*) is the number of molecules at time *t*, after an interval Δ*t* the number at *t* + Δ*t* will be *N*(*t*) + (*N*(*t* + Δ*t*) − *N*(*t*)) = *N*(*t* + Δ*t*). If synthesis is stopped after a pulse of length *t*_*p*_, the number of labeled molecules that can be found at *t*_*p*_ is given by *N*(*t*_*p*_). Let us define *N*′(*t*_*p*_ + Δ*t*) as the number of molecules at *t*_*p*_ + Δ*t*, if we stop synthesis at *t*_*p*_. This quantity is different from *N*(*t*_*p*_ + Δ*t*), which is the number of molecules one would have had if labeling had not stopped at time *t*_*p*_. Thus, *N*′(*t*_*p*_ + Δ*t*) is given by

$$\begin{array}{c} N^{\prime }\left(t_{p}+\Delta t\right)\;=\;N\left(t_{p}\right)+\left[\left(N\left(t_{p}+\Delta t\right)-N\left(t_{p}\right)\right)-N\left(\Delta t\right)\right]\;, \end{array}$$ (8)

because the molecules *N*(Δ*t*) that would have been delivered are simply missing. During the chase phase, thus, the relative amount of labeled molecules must decrease according to

$$\begin{array}{c} C\left(t_{p}+\Delta t\right)\;=\;\frac{N^{\prime }\left(t_{p}+\Delta t\right)}{N(t_{p})}=\;\frac{N\left(t_{p}+\Delta t\right)-N\left(\Delta t\right)}{N(t_{p})\;}. \end{array}$$ (9)

If labeling is stopped at steady state, the relative amount of labeled molecules is obtained by Eq (9) by setting *t*_*p*_ → ∞. An explicit formulation of Eq (9) is finally obtained substituting Eq (7)

$$\begin{array}{c} C\left(\Delta t\right)\;=\;\frac{1}{N(t_{p})}\int_{\Delta t}^{t_{p}+\Delta t}\left(1-F_{{}_{T}}\left(u\right)\right)du\;, \end{array}$$ (10)

where the denominator just ensures that *C*(0) = 1 and Δ*t* represents the time of measurement after the end of the pulse. If *f*_*T*_ is an exponential probability density, *C*(Δ*t*) decays also exponentially with the same rate independently of the pulse length.

### Data analysis

Once *F*_*T*_ is known, the decay pattern during the chase phase is given by *C*(Δ*t*) from Eq (9). In most of the cases, however, only the *experimental* values of $\tilde{C}$ at different time points Δ*t* = Δ_1_, Δ_2_, … , Δ_*n*_ are available. Thus one defines a parametric form of *F*_*T*_ and uses the data to fit the parameters that minimize the function

$$\begin{array}{c} M\;=\;\sum_{j=1}^{n}{\left(\ln\left(C\left(\Delta_{j}\right)\right)-\ln\left(\tilde{C}\left(\Delta_{j}\right)\right)\right)}^{2}\;, \end{array}$$ (11)

the reason for the log being that one can generally assume a lognormal distribution of the measurement error. When data from *m* experiments on the same molecule using different pulse durations *t*_*p*_ = *t*_1_, … , *t*_*m*_ are available, the function to minimize becomes

$$\begin{array}{c} M\;=\;\sum_{p=1}^{m}\sum_{j=1}^{n}{\left(\ln\left(C\left(\Delta_{j}\right)\right)-\ln\left(\tilde{C}\left(\Delta_{j}\right)\right)\right)}^{2}\;, \end{array}$$ (12)

where in principle, the Δ_*j*_ might also be different from one experiment with one pulse length and another experiment with a different pulse length.

### Model calibration (parameter estimation)

We use several functions in MATLAB^®^ to calibrate the models. To minimize the objective function, we use fmincon (bounds: *κ*_10_, *κ*_12_, *κ*_20_ ∈ [0.000001,1]). We also used GlobalSearch (with the default settings) and MultiStart (1000 start points) to better sample the available parameter space.

## Results and Discussion

**Fig 2:**
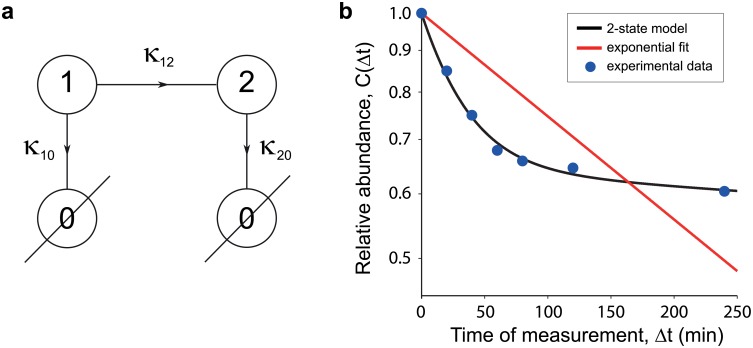
Markov chain representation of a Markov process and 2-state model fit to a decay curve. **(a)** Markov chain representation of a degradation process. Biochemical pathways (such as degradation) can be readily translated into Markov chain models: each biochemical entity is represented as a Markov state (circles) and the reaction speeds are represented as fluxes between the states (arrows). Here we show a possible Markov model of degradation containing two states. Newly synthesized molecules are in state 1. From state 1, there are two possible paths, either to state 2 with rate *κ*_12_ or degraded with rate *κ*_10_. For those molecules that reach state 2, they are degraded with rate *κ*_20_. The rates *κ*_10_ and *κ*_20_ are necessarily different, otherwise the model collapses into a 1 state model. **(b)** 2-state model fit (black line) and exponential fit (red line) to sample data with 1 minute pulse (data from [9], blue spots). Note that in the log(abundance)-linear(time) scale, the data does not resemble a straight line, thus necessitating a model more complicated than a single exponential. The best fit using Eq (11) with *C*(Δ*t*) from Eq (16) gives the following parameters: *κ*_10_ = 0.0109 min^ − 1^, *κ*_20_ = 0.002 min^ − 1^, *κ*_12_ = 0.0189 min^ − 1^, and pulse = 1 min. *κ*_*exp*_ = 0.0029. The decision in favor of the two-stage model is made on the basis of the AIC criterion thanks to its very small RSS.

Most mRNA and protein decay patterns can be fit with one of two simple models: A two-stage model (Fig 2a) always results in a better fit than a one-stage (exponential decay) model, but many patterns are sufficiently described by the simpler one-stage model. The decision of whether a two-stage model or the exponential fit should be adopted depends on the balance between accuracy of the fit and number of parameters. The Akaike Information Criterion (AIC) is one such measure that can be used to select the better model [23]. In this paper, we concentrate on the use of the two-stage model. The motivation for this is two fold—firstly, for decay patterns from one-stage models (exponential decay), one does not need to take the pulse length into account. The decay curve of the system with exponential decay is independent of the pulse. Secondly, in the examples that we will later present, the one-stage model does not adequately capture the dynamics of the system (Fig 2b). Through the AIC, we find that one-stage models are too simplistic, and three stage models are not adequately identifiable. For other systems with degradation patterns not well described by a two-stage Markov state model, Eq (10) can be used to derive the appropriate expression. The two-stage model applied to the data of Ref. [9] has an RSS of 0.0011 (Fig 2b). To evaluate the goodness of fit, we apply the chi-squared test with three d.o.f. and find that we can not reject the null hypothesis which is that the two-stage model is a good fit (p-value < 10^ − 5^).

The general mathematical methodology to describe the decay (see Methods) requires a specific form of the lifetime distribution in order to become practically useful. Ideas from biochemical networks indicate that a Markov chain is a useful and flexible framework to develop the basic models of molecular degradation [8, 21]. In terms of a Markov chain the two-stage model consists of two states, say state 1 and state 2, and a degraded state called state 0 connected to each other (Fig 2a). The rates *κ*_12_, *κ*_10_, and *κ*_20_ govern the transitions from state 1 to state 2, from state 1 to state 0, and from state 2 to state 0, respectively. In addition, the rates *κ*_10_, and *κ*_20_ are composed of the degradation rates from each state and the basal dilution rate due to cell division and population growth; the basal dilution rate can have a negligible effect if the timescale of the experiment is shorter than the doubling time of the cell culture.

Using this network we model the life of a single molecule as follows. The molecule is synthesized to be in biochemical state 1 (or it moves very quickly through a series of biochemical steps until it reaches a biochemical state that we call state 1). The molecule dwells for a random amount of time in state 1. The average amount of time spent in state 1 is *τ*_1_ = 1/(*κ*_10_ + *κ*_12_). The molecule then leaves state 1 and is either degraded, *i.e.* it jumps to state 0 with probability *P*_10_ = *τ*_1_ *κ*_10_, or it jumps into biochemical state 2 with probability *P*_12_ = *τ*_1_ *κ*_12_. If the molecule attains state 2, it will dwell in this state for a random amount of time with average *τ*_2_ = 1/*κ*_20_ until it is eventually degraded (*i.e.* *P*_20_ = 1). The two-stage model does not mean that there are only two biochemical states for a molecule, it says that all other states are visited very quickly and that, at the end, two compound states are sufficient to describe the decay pattern for that molecule.

The lifetime of the molecule is now just the random time required to move from state 1 to state 0, taking into account the possibility to visit state 2 before degradation. Technically, this is the absorption time in 0 starting from state 1 and its probability density is the lifetime probability density *f*_*T*_(*t*). The equivalence of lifetime and absorption time is useful because there are plenty of mathematical tools to compute the probability density of the time to absorption. Once the probability density *f*_*T*_(*t*) is computed in terms of the (yet unknown) rates *κ*_10_, *κ*_12_ and *κ*_20_, and of the pulse length *t*_*p*_, it can be used to compute the relative abundance *C*(Δ*t*) (Eq 10). Fitting the data will then finally deliver the appropriate values of the rates (Fig 2b).

The beauty of the two-stage model is that finding the distribution of the lifetime *T* is simple. If *ρ*_1_(*t*) and *ρ*_2_(*t*) are the dwell time distributions on states 1 and 2, respectively, then it results that

$$\begin{array}{c} F_{T}\left(t\right)\;=\;P_{{}_{10}}\int_{0}^{t}\rho_{1}\left(\tau \right)d\tau +P_{{}_{12}}\int_{0}^{t}\rho_{1}\left(\tau \right)\int_{0}^{t-\tau }\rho_{2}\left(u\right)du\;d\tau \;, \end{array}$$ (13)

with *P*_10_ and *P*_12_ defined earlier. In a Markov chain, the distributions are explicitly given by

$$\begin{array}{c} \begin{array}{ccc} \rho_{1}\left(t\right) & = & \left(\kappa_{{}_{10}}+\kappa_{{}_{12}}\right)\exp\left(-(\kappa_{{}_{10}}+\kappa_{{}_{12}})t\right)\\ \rho_{2}\left(t\right) & = & \kappa_{{}_{20}}\exp\left(-\kappa_{{}_{20}}t\right)\;. \end{array} \end{array}$$ (14)

Working through the formulas to obtain *C*(Δ*t*) is now a simple matter of calculus (see Eq 10 and S1 Supporting Information). The final result is a formula depending on three unknown parameters (*κ*_10_, *κ*_12_ and *κ*_20_) and the known parameter *t*_*p*_ to be used to fit any decay curve.

After defining the two factors

$$\begin{array}{l} A_{c}=\frac{\kappa_{10}-\kappa_{20}}{\kappa_{10}+\kappa_{12}}\left[1-\text{exp}(-(\kappa_{10}+\kappa_{12})t_{p})\right]\\ B_{c}=\frac{\kappa_{12}}{\kappa_{20}}\left[1-\text{exp}(-\kappa_{20}t_{p})\right], \end{array}$$ (15)

we obtain the relative abundance

$$\begin{array}{c} C\left(\Delta t\right)\;=\;\frac{A_{c}}{A_{c}+B_{c}}\exp\left(-(\kappa_{{}_{10}}+\kappa_{{}_{12}})\Delta t\right)+\frac{B_{c}}{A_{c}+B_{c}}\exp\left(-\kappa_{{}_{20}}\Delta t\right)\;, \end{array}$$ (16)

which is a single formula to fit the data for any pulse duration *t*_*p*_. At steady state, when *t*_*p*_ → ∞, the two exponential functions in Eq (15) disappear. For a finite *t*_*p*_, instead, the two factors *A*_*c*_ and *B*_*c*_ get rescaled in a asymmetric fashion by the effect of the pulse. In the ideal case of a pulse of zero duration, *i.e.* *t*_*p*_ → 0, we would have *C*(Δ*t*) = 1 − *F*_*T*_(Δ*t*). In the limit when decay is exponential, instead, *C*(Δ*t*) is an exponential function independent of *t*_*p*_.

### Examples

**Fig 3:**
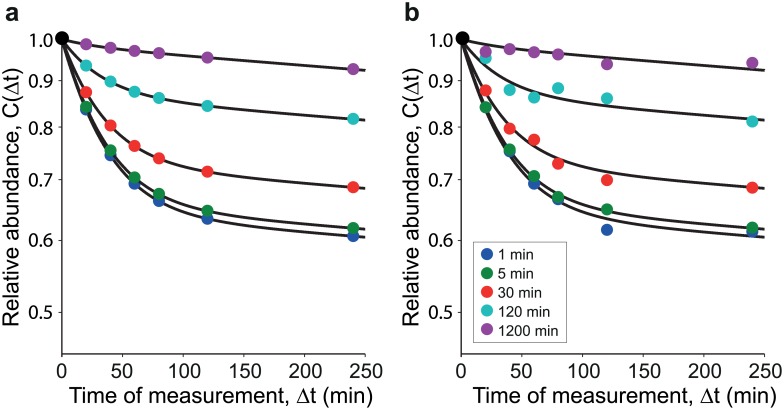
Model calibration with fabricated data. **(a)** Verification of fitting procedure using simulated data separately. Using the parameters obtained from the best fit model to the data from [9] for pulse = 1 min, we fabricate sample data by calculating the abundance over time (dots) for different pulse lengths (1, 5, 30, 120, 1200 minutes) using the function that gives the decay pattern of the relative abundance *C*(Δ*t*), Eq (16), as function of the measurement time Δ*t*. We then fit resultant decay patterns with our fitting routine. We get back the same rates that were used to simulated the data for each experiment (Table 1). This shows that if the system in the background is unchanging, we can reliably extract the parameters of the system by fitting the decay patterns individually. *κ*_10_ = 0.0109 min^ − 1^, *κ*_20_ = 0.002 min^ − 1^, and *κ*_12_ = 0.0189 min^ − 1^. **(b)** Simultaneous fit of pooled simulated data. Here the simulated data is augmented with a small amount of multiplicative noise, Eq (17). We fit the whole collection of data simultaneously (see Methods). Values very close to our original simulation parameters are obtained (Table 1 last row). This shows that under steady experimental conditions, we can reliably extract the parameters of the system by fitting the decay patterns simultaneously.

With Eq (16) we have been able to determine a single mathematical formula that describes the whole decay pattern. The next challenge is to show that the fitting procedure (Methods) works. To show this, we proceed as follows. We have first extracted the decay patterns from Ref. [9], where the decay of proteins content of HeLa cells was monitored after pulse of duration 1, 5, 30, 120, 1200 minutes. We have taken the decay pattern corresponding to a 1 minute pulse and found the best fit for the three rates, *κ*_10_ = 0.0109 min^−1^, *κ*_20_ = 0.0002 min^−1^ and *κ*_12_ = 0.0189 min^−1^ (Fig 2), which correspond to an average lifetime $\bar{T}$ of more than 53 hours, see Eq E in S1 Supporting Information. We have then plugged these rates in Eq (16) to fabricate artificial datasets corresponding to 1, 5, 30, 120, 1200 minutes pulse and fit them to prove that the fit gives back the same rates used to fabricate the data. Contrary to the fits to the experimental data 2, the result of the fit (Fig 3a) gives rates that are identical to those used to generate the data (Table 1). This is good news because we want to be sure that the procedure is self-consistent. We have also added some noise in the form of

$$\begin{array}{c} C^{\left(\text{noise}\right)}\left(\Delta t\right)\;=\;C\left(\Delta t\right)\exp\left(\epsilon \zeta \right)\;, \end{array}$$ (17)

for Δ*t* > 0, *ϵ* = 0.01, and *ζ* ∈ [0,1) is a uniformly distributed random number which is drawn independently for each Δ*t*. Adding the noise is important to demonstrate the robustness of the fitting procedure and the sensitivity of the fitting function to small perturbations. In fact, the fit of the single fabricated decay patterns deteriorates as the pulse length increases depending on the amount of noise, probably because at long pulses state 2 dominates the behavior of the decay pattern. If the most populated state is state 2, as we have in our example, it becomes more and more difficult to detect events occurring in state 1 from the measurements as the pulse duration increases. This indicates that pulses of short duration should be preferred. Nevertheless, when we search for the three parameters to fit all pulsed curves at once, the procedure becomes quite reliable and the fit quality is very high (Fig 3b and Table 1 last row).

**Table 1:** Model parameters for simulated data with different pulse lengths. Parameters from the best fit to the data simulated with different pulse lengths and the 2-state model as found by Multistart (MATLAB^®^) with 1000 start points. Minimization by fmincon (bounds *κ*_10_, *κ*_20_, *κ*_12_ ∈ [0.000001,1]). Notice that the fits yield results identical to those used to generate the data. This proves the self-consistency of the procedure. Conversely in Table 2, the parameter values are not stable; suggesting different degradation system dynamics in each experiment.

pulse	*κ*_10_ (min^ − 1^)	*κ*_20_ (min^ − 1^)	*κ*_12_ (min^ − 1^)
simulation values	0.0109	0.0002	0.0189
1 min	0.0109	0.0002	0.0189
5 min	0.0109	0.0002	0.0189
30 min	0.0109	0.0002	0.0189
120 min	0.0108	0.0002	0.0189
1200 min	0.0139	0.0002	0.0221
whole collection of data + noise	0.0050	0.0003	0.0254

**Fig 4:**
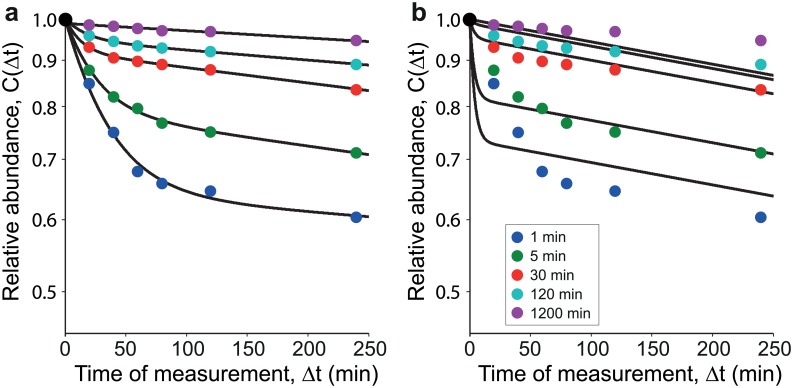
Model calibration with data from Ref. [9]. **(a)** Decay patterns from Ref. [9] fit individually. Each decay pattern from Ref. [9] is fit with the 2-state model using Eq (11) with *C*(Δ*t*) from Eq (16). We find that for some of the decay patterns, the parameters obtained from the fitting are different from the others (Table 2). This implies that the underlying system has changed in the different experiments. Possibly the labeling procedure has affected the cells and contributed to a change in the internal environment. **(b)** Simultaneous fit of pooled real data with Eq (12). Here we pool the decay patterns and fit them simultaneously with the 2-state model. No good fits were found, despite using global and multistart techniques in the parameter search process. This implies that the underlying systems across the experiments can not be described by one unified model, at least not the two state model that we have considered. Possibly the labeling procedure has affected the cells and contributed to a change in the internal environment.

As a next step, we have fit the individual experimental decay patterns as provided in Ref. [9] (Fig 4a) and compared the rates with each other (Table 2). We find that the rates depart from each other much more than those found with the fabricated data. With the proviso that we have extracted the data from an old low-definition figure in semilogarithmic scale, the differences in the rates definitively increase with the duration of the pulse. Also the search for a unique set of rates that allow fitting the various pulsed curves all together gives a poor result (Fig 4b). The most likely explanation of the wrong fit is that the labeling has affected the cells and has thus contributed to change its internal environment so that protein degradation after a long pulse is different than protein degradation after a short pulse, an effect that may certainly depend also on the labeling technique.

**Table 2:** Model parameters for data from Ref. [9]. Parameters from the best fit to the 2-state model as found by Multistart (MATLAB^®^) with 1000 start points. Minimization by fmincon (bounds *κ*_10_, *κ*_20_, *κ*_12_ ∈ [0.000001,1] min^ − 1^). Notice that each fit yields different parameters. This suggests that the degradation system dynamics in each experiment is different. In contrast Table 1 shows that the fits to simulated data with consistent parameters return the same rates. Data are reported in Table A in S1 Supporting Information

pulse	*κ*_10_ (min^ − 1^)	*κ*_20_ (min^ − 1^)	*κ*_12_ (min^ − 1^)
1 min	0.0109	0.0002	0.0189
5 min	0.0091	0.0004	0.0294
30 min	0.0139	0.0004	0.0610
120 min	0.0172	0.0003	0.0434
1200 min	0.0759	0.0002	0.0451

### Just pulse, no chase

So far, we have focused our derivations on experiments where marked molecules are synthesized during a pulse of synthesis, and measurements of the marked molecules are taken after the chasing period. Another experimental set up is to have the pulse period up until the time of measurement, without “chase” [24, 25]. The steady state value, *N*^(st)^, is a convenient value to use for normalizing the measurements. Thus, the ratio

$$\begin{array}{c} P\left(\Delta t\right)=\frac{N(t)}{N^{\left(\text{st}\right)}}\;, \end{array}$$ (18)

is a convenient quantity for the relative abundance of labeled molecules. Here, Δ*t* is the time of measurement from the start of labeling. Using the same framework, from Eq (7) and Eq D in S1 Supporting Information we obtain

$$\begin{array}{c} P\left(\Delta t\right)=\frac{A_{p}}{A_{p}+B_{p}}\left(1-\exp(-\left(\kappa_{{}_{10}}+\kappa_{{}_{12}}\right)\Delta t)\right)+\frac{B_{p}}{A_{p}+B_{p}}\left(1-\exp(-\kappa_{{}_{20}}\Delta t)\right)\;, \end{array}$$ (19)

where the two factors *A*_*p*_ and *B*_*p*_ are given by

$$\begin{array}{c} A_{p}=\kappa_{20}(\kappa_{10}-\kappa_{20})\\ B_{p}=\kappa_{12}(\kappa_{10}+\kappa_{12}), \end{array}$$ (20)

as the expression for *P* according to a two-stage model.

If the steady state measurement of molecules is not available, any other time point can also be used. With other time points used as normalization, the expression for *P* is more complicated, but still solvable by hand for the two-stage model.

**Fig 5:**
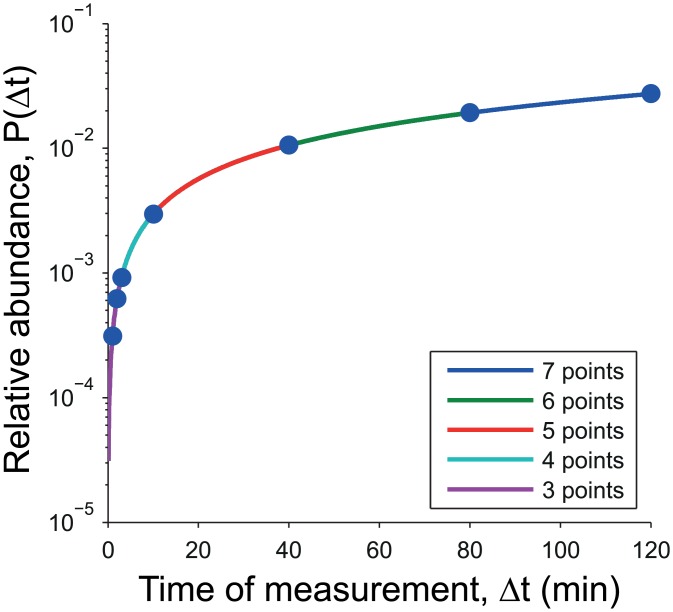
Simulation of pulse no chase experiments. Simulation of pulse no chase experiments, where the pulse is applied up until the time of measurement. Data is produced with rates *κ*_10_ = 0.0109 min^−1^, *κ*_20_ = 0.0002 min^−1^ and *κ*_12_ = 0.0189 min^−1^ using Eq (19). Traces show the results of several fits, each fitting taking into consideration one additional data point (Table 3).

One might assume that with more data, we would see an improvement in the determination of the model parameters. Initially we see this is true, but once we have taken data up to t = 20–40 minutes (4–5 points), additional sampling does not significantly improve the fit (Fig 5 and Table 3). This can be explained by examining the sensitivities of the output to the parameters. We define the sensitivity of the output to parameter *κ*_*i*_ as

$$\begin{array}{c} v_{\kappa_{i}}\left(\kappa_{i},t\right)=\frac{dP(t)}{d\kappa_{i}} \end{array}$$ (21)

This equation describes the amount that the output should change if the values of *κ*_*i*_ and time change. The expressions are quite lengthy, but we can write them for each of the parameters in the system in closed form. By plugging in the “true” values for *κ*_*j* ≠ *i*_ we can plot the sensitivities as a function of time and *κ*_*i*_.

**Table 3:** Model parameters for simulated pulse no chase data. Parameters from the best fit to the 2-state model as found by Multistart (MATLAB^®^) with 100 start points. Minimization by fmincon (bounds *κ*_10_, *κ*_20_, *κ*_12_ ∈ [0.000001,1] min^ − 1^). The first row is the best fit for the data as if the experimentalist only took 3 measurements at *t* = 1,2,3 minutes. The 2nd row takes one more measurement (*t* = 10 minutes) into consideration.

	*κ*_10_ (min^ − 1^)	*κ*_20_ (min^ − 1^)	*κ*_12_ (min^ − 1^)
simulation values	0.0109	0.0002	0.0189
3 points	0.0104	0.0002	0.0240
4 points	0.0119	0.0002	0.0402
5 points	0.0109	0.0002	0.0189
6 points	0.0109	0.0002	0.0190
7 points	0.0109	0.0002	0.0189

**Fig 6:**
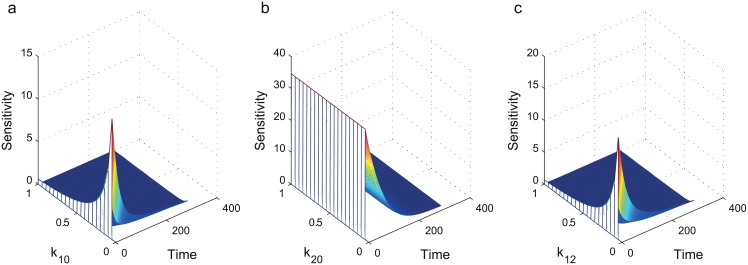
Sensitivity of *P*(Δ*t*) to the parameters *κ*_10_, *κ*_20_ and *κ*_12_. We plot the sensitivities of the measurements in a pulse no chase experiment assuming a 2-state model with *κ*_10_ = 0.0109 min^ − 1^, *κ*_20_ = 0.0002 min^ − 1^ and *κ*_12_ = 0.0189 min^ − 1^. **(a)** Output is only sensitive to small values of *κ*_10_. **(b)** Output is sensitive to a range of *κ*_20_. **(c)** Output is only sensitive to small values of *κ*_12_. For all parameters, taking more measurements at later timepoints does not help the parameter estimation because the output is not sensitive to deviations in the parameters at late times.

We discover that the output at late time points are insensitive to the parameters. Furthermore, the output is only sensitive to small values of *κ*_10_ and *κ*_12_ (Fig 6). Note that here we have calculated the local sensitivities—the result is likely to differ for different ranges of *κ*’s.

### Dynamical properties

The half-time *t*_1/2_ is traditionally used as a quantity to measure the stability of the molecules. Unfortunately, this quantity is meaningful only when the decay is exponential because in that case there is a universal relationship between the half-time, the rate of degradation and the average lifetime. In a non-exponential decay, the universality of the relationship is lost. For these systems, the average lifetime $\bar{T}$ is a more meaningful quantity. Indeed, the steady state quantity of molecules (assuming stationary growth conditions and non-synchronized cells) is given by

$$\begin{array}{c} N^{\left(\text{st}\right)}\;=\;\omega \bar{T}\;, \end{array}$$ (22)

(Methods) thus allowing, at least in principle, to estimate the synthesis rate *ω* once the steady state amount of molecules per cell has been determined.

Once the rates *κ*_*ij*_ have been estimated, a number of other properties can be easily computed. The average time spent on state 1 is given by *ν*_1_ = 1/(*κ*_10_ + *κ*_12_). The fraction of molecules able to pass from state 1 to state 2 is given by *κ*_12_ *ν*_1_. The molecules able to reach state 2 will occupy this state for an average amount of time given by *ν*_2_ = 1/*κ*_20_. Finally, the fractions *π*_1_ and *π*_2_ of molecules found in states 1 and 2 is given by

$$\begin{array}{c} \pi_{1}\;=\;\frac{\kappa_{{}_{20}}}{\kappa_{{}_{12}}+\kappa_{{}_{20}}}\;\;\;\text{and}\;\;\;\pi_{2}\;=\;1-\pi_{1}\;. \end{array}$$ (23)

Furthermore, if for any reason the half-time *t*_1/2_ is sought, it can be computed numerically by inverting the equation

$$\begin{array}{c} C_{\infty }\left(t_{1/2}\right)\;=\;\frac{1}{2}\;, \end{array}$$ (24)

where *C*_∞_ is the expression given in Eq (16) when *t*_*p*_ → ∞. Notice that the use of Eq (24) for any finite *t*_*p*_ < ∞ would lead to an apparent the half-time different from the true value *t*_1/2_ thus making the apparent half-time dependent on the length of the pulse.

## Conclusion

Even if steady state expression levels of molecules depend only on their average lifetime, non-exponential decay has an effect concerning timing and dynamics of cellular response [18, 19, 26], and cell-to-cell variation in cellular content when cultures are subject to stochastic effects [27]. For this reason, it is an aspect of regulation that has to be taken into account if one wants to understand the reaction of cells to stress or to environmental changes. Furthermore, disentangling various hypotheses concerning the nature and the structure of biochemical pathways responsible for degradation [10, 11, 21] is possible only when models take the complexity of the pathways into account. Nevertheless, the derivation of the average lifetime required to compute the steady state properties (and the synthesis rate, if an independent measurement of the steady state abundance is available) requires the correct mathematics, which in most of the cases is not given by the exponential fit.

In this manuscript we focused on the two-stage model as a good approximation that describes most of the non-exponential decay patterns. This generalization is based on extensive experience from fitting thousands of mRNA and protein decay patterns. Nevertheless, there is no reason in principle to restrict the number of states to two, since the number of biochemical steps related to the destabilization of molecules is conceivably much larger. While it is not difficult to draw and mathematically describe larger networks with more states and alternative pathways, there is rarely enough data available to fix all the parameters [23]. A lucky exception is provided by sets of experiments where measurements are taken with different parts of the biochemical network deactivated [10, 21].

Pulse-chase experimental techniques offer the advantage of a low impact on the metabolism and well-being of the cells. Yet, little attention has been devoted to the fact that the pulse has an effect on the decay pattern if the decay pattern is not exponential. By working through the mathematics of single molecule decay, we derive and demonstrate in a novel approach a series of equations that anyone can use to fit complex decay patterns. The values of the rates would then finally provide a valuable tool to compute other dynamic quantities.

When assessing the nature of the decay pattern we recommend to compare the fit of the two-stage model with the simpler exponential model to ensure that the data supports the more complex model. As a guideline, long pulses (and thus steady state measurements) tend to obfuscate short term dynamics and thus appear as exponential decay, as this catches the long term dynamics of the degradation process. We recommend the use of the shortest pulse as possible in order to detect short time effects. More pulses of different lengths increase the robustness and our confidence in the model and its calibration. As a byproduct, fitting curves generated from pulses of different lengths may allow discovery of perturbing effects from labeling, when the fits of the individual curves reveal very different rates beyond what one would expect from noise. One caveat to the current approach outlined here is the assumption that the quantities measured are reflective of synthesis and degradation only. However, in in vivo systems, each cell division results in a dilution effect of the molecules. Thus for such experiments it is imperative to choose experimental times well within the cell division time, or take cell division into account.

## Supporting Information

S1 Supporting Information Supplementary note. The file contains some supplementary calculations and the table with the experimental data extracted from Ref. [9].
(PDF)
