# Supplementary material for: Degradation Parameters from Pulse-Chase Experiments
Source: PLoS One. 2016 May 16;11(5):e0155028. doi: 10.1371/journal.pone.0155028 (PMC4868333; doi:10.1371/journal.pone.0155028)
Supplement: S1 Supporting Information — The file contains some supplementary calculations and the table with the experimental data extracted from Ref. [9]. (PDF) [file pone.0155028.s001.pdf]

Supporting Information
Degradation Parameters from Pulse-Chase Experiments

Celine Sin, Davide Chiarugi, Angelo Valleriani*
Department of Theory and Bio-Systems
Max Planck Institute of Colloids and Interfaces
14424 Potsdam, Germany

Supplementary Section 1. Integrals

For the two-stage model introduced in section *Results* there are a few minor integrals, that may become interesting in certain applications. Using F_T from Eq. (13) and the $\rho_i(t)$ from Eqs. (14) we obtain

$$F_T(t) = 1 - \frac{(\kappa_{10} - \kappa_{20})e^{-(\kappa_{10}+\kappa_{12})t} + \kappa_{12}e^{-\kappa_{20}t}}{\kappa_{10} + \kappa_{12} - \kappa_{20}}\,, \tag{A}$$

from which the probability density is given by

$$f_T(t) \;=\; \frac{(\kappa_{10} - \kappa_{20})(\kappa_{10} + \kappa_{12})e^{-(\kappa_{10}+\kappa_{12})t} + \kappa_{12}\kappa_{20}e^{-\kappa_{20}t}}{\kappa_{10} + \kappa_{12} - \kappa_{20}}\,, \tag{B}$$

and the age-dependent degradation rate is also given by

$$\delta(a) \;=\; \frac{(\kappa_{10} - \kappa_{20})(\kappa_{10} + \kappa_{12})e^{-(\kappa_{10}+\kappa_{12})a} + \kappa_{12}\kappa_{20}e^{-\kappa_{20}a}}{(\kappa_{10} - \kappa_{20})e^{-(\kappa_{10}+\kappa_{12})a} + \kappa_{12}e^{-\kappa_{20}a}}\,. \tag{C}$$

Notice that $\delta(a)$ becomes a constant in the limit of an exponential decay, obtained either when $\kappa_{12} \to 0$ or when $\kappa_{10} = \kappa_{20}$. Central to the derivation of $C(\Delta t)$ and $P(\Delta t)$ is the following integral

$$\int_{\Delta t}^{t_p+\Delta t} (1 - F_T(t))\,\mathrm{d}t = \frac{A_c e^{-(\kappa_{10}+\kappa_{12})\Delta t} + B_c e^{-\kappa_{20}\Delta t}}{\kappa_{10} + \kappa_{12} - \kappa_{20}}\,, \tag{D}$$

where A_c and B_c, given in Eqs. (15), contain the dependence on the pulse duration t_p. This integral, allows us to finally derive the average lifetime of the degrading molecules

$$\overline{T} \;=\; \frac{\kappa_{12} + \kappa_{20}}{\kappa_{20}(\kappa_{10} + \kappa_{12})}\,. \tag{E}$$

*email: `angelo.valleriani@mpikg.mpg.de`

Supplementary Section 2. Experimental data from Ref. [1]

Measurement time \ Pulse duration	1 min	5 min	30 min	120 min	1200 min
0 min	100	100	100	100	100
20 min	84.9067	87.7513	93.0551	95.8508	98.525
40 min	74.9383	82.043	90.6058	94.4322	98.1468
60 min	67.8501	79.6414	89.7735	93.4221	97.5927
80 min	65.8373	76.7611	89.0894	92.8671	97.0384
120 min	64.5597	75.0701	87.871	91.9931	96.8331
240 min	60.44	71.1759	83.5673	89.0805	94.6325

Supplementary Table A: **Data from Ref. [1]** Each column shows the time course measurements after pulse time of 1, 5, 30, 120, 1200 minutes. Measurements are taken at t = 0, 20, 40, 60, 80, 120 minutes.

References

[1] Wheatley DN, Giddings MR, Inglis MS. Kinetics of degradation of "short-" and "long-lived" proteins in cultured mammalian cells. Cell biology International Reports. 1980;4(12):1081–1090.
